# Supplementary material for: Direct determination of high-order transverse ligand field parameters via µSQUID-EPR in a Et4N[160GdPc2] SMM
Source: Nat Commun. 2023 Jun 8;14:3361. doi: 10.1038/s41467-023-39003-5 (PMC10250539; doi:10.1038/s41467-023-39003-5)
Supplement: Supplementary file 1 — Supplementary Information [file 41467_2023_39003_MOESM1_ESM.pdf]

Direct Determination of High-Order Transverse Ligand Field Parameters *via* µSQUID-EPR in a $Et_4N[^{160}GdPc_2]$ SMM

Gheorghe Taran[a], Eufemio Moreno-Pineda[b], Michael Schulze[a], Edgar Bonet[c], Mario Ruben*[d,e,f] and Wolfgang Wernsdorfer*[a,f]

a. *Physikalisches Institut, Karlsruhe Institute of Technology, D-76131 Karlsruhe, Germany.*
b. *Depto. de Química-Física, Facultad de Ciencias Naturales, Exactas y Tecnología, Universidad de Panamá, Panamá.*
c. *Néel Institute, CNRS, 25 rue des Martyrs, Grenoble 38042, France.*
d. *Centre Européen de Sciences Quantiques (CESQ) within the Institut de Science et d'Ingénierie Supramoléculaires (ISIS), 8 allée Gaspard Monge, BP 70028, 67083 Strasbourg Cedex France.*
e. *Institute of Nanotechnology (INT), Karlsruhe Institute of Technology (KIT), Hermann-von-Helmholtz-Plats 1, D-76344 Eggenstein-Leopoldshafen, Germany.*
f. *Institute for Quantum Materials and Technology (IQMT), Karlsruhe Institute of Technology (KIT), Hermann-von-Helmholtz-Platz 1, D-76344 Eggenstein-Leopoldshafen, Germany.*

*Correspondence to: eufemio.moreno@up.ac.pa; mario.ruben@kit.edu; wolfgang.wernsdorfer@kit.edu

Experimental Details

Unless stated otherwise, all reagents and solvents were used without further purification. The isotopically enriched gadolinium oxide starting material, $^{160}Gd_2O_3$ (98.3%), was purchased from BuyIsotope, Sweden. The oxide lanthanide was then converted to the acetylacetonate counterparts employing published procedures.[1] Column chromatography was carried out using Silica 60 Å (particle size 35-70 µm, Fisher, UK) as the stationary phase, and TLC was performed on precoated silica gel plates (0.25 mm thick, 60 F254, Merck, Germany).

A. Synthetic Method

A.1. General procedure for the synthesis of $Ln(C_{32}H_{16}N_8)_2$: To a solution of 1,2-dicyanobenzene (563 mg, 4.4 mmol) and the respective lanthanide acetylacetonate hydrate (0.6 mmol) in hot hexanol (5 mL) were added 1,8-Diazabicyclo[5.4.0]undec-7-ene (DBU, 0.33 mL) and $BzMe_3NCl$ (0.33 mg, 1.8 µmol) and the reaction mixture heated under reflux (157 °C) for 48 h. During this time, the colour changed from yellow to dark green. After 48 h, the reaction mixture was cooled to room temperature and the slurry was added to hexanes (100 mL). The precipitated was collected by filtration and washed with several portions of Et_2O. The precipitated was posteriorly separated *via* column chromatography employing dichloromethane as eluent, resulting in a green material. The compound can be precipitated upon addition of hexanes yielding the microcrystalline the lanthanide di-phthalocyanine $LnPc_2$ complex (where Ln = Y and ^{160}Gd). For **$Y(C_{32}H_{16}N_8)_2$**: ESI-MS m/z, calcd. (found): 1113.2054 (1113.1223) (negative ionization); Elemental analysis calcd. (found): C = 69.01 % (68.98 %), H = 2.89 % (2.87%), N = 20.12 % (20.08 %). For **$^{160}Gd(C_{32}H_{16}N_8)_2$**: ESI-MS m/z, calcd. (found): 1184.2266 (1184.2416) (negative ionisation). Elemental analysis, calcd. (found): C = 65.02 % (64.91 %), H = 2.73 % (2.68 %), N = 18.96 % (18.87 %).

A.2. $Et_4N[Y(C_{32}H_{16}N_8)_2]$ and $Et_4N[^{160}Gd(C_{32}H_{16}N_8)_2]$: To a suspension of the neutral lanthanide double-decker (0.042 mmol) in methanol (5 mL) under argon atmosphere, was added tetraethylammonium iodide (11 mg, 0.08 mmol) and hydrazine (0.2 mL, 64%) in methanol (5 mL). The slurry was stirred overnight and posteriorly filtered affording the negatively-charged **$Et_4N[Y(C_{32}H_{16}N_8)_2]$** and **$Et_4N[^{160}Gd(C_{32}H_{16}N_8)_2]$**. For **$Et_4N[Y(C_{32}H_{16}N_8)_2]$**: ESI MS m/z, calcd. (found): 1113.2054 (1113.1237) (M^-, negative ionization); Elemental analysis calcd. (found) for **$Et_4N[Y(C_{32}H_{16}N_8)_2]$**: C = 69.5 % (69.32 %), H = 4.21% (4.28 %), N = 19.14 % (19.21 %). For **$Et_4N[^{160}Gd(C_{32}H_{16}N_8)_2]$**: ESI-MS m/z, calcd. (found): 1184.2266

(1184.2308) (negative ionisation). Elemental analysis, calcd. (found): C = 65.88 % (64.93 %), H = 3.99 % (4.02 %), N = 18.14 % (18.17 %).

A.3. Diluted complex 5%$Et_4N[^{160}Gd(C_{32}H_{16}N_8)_2]$@95%$Et_4N[Y(C_{32}H_{16}N_8)_2]$: The magnetically dilute sample composed of 5% $Et_4N[^{160}Gd(C_{32}H_{16}N_8)_2]$ and 95% $Et_4N[Y(C_{32}H_{16}N_8)_2]$ was obtained by combining accurately measured amounts of $Et_4N[^{160}Gd(C_{32}H_{16}N_8)_2]$ and $Et_4N[Y(C_{32}H_{16}N_8)_2]$ in 5:95 molar ratio. The compound was then solubilized in hot acetone and recrystallized by slow evaporation, affording very regular block tetragonal red crystals.

B. Crystallography

Single crystal X-ray diffraction data of **$Et_4N[^{160}Gd(C_{32}H_{16}N_8)_2]$** and **$Et_4N[Y(C_{32}H_{16}N_8)_2]$** was collected employing STOE StadiVari 25 diffractometer with a Pilatus300 K detector using GeniX 3D HF micro focus with MoKα radiation (λ = 0.71073 Å). The structure was solved using direct methods and was refined by full-matrix least-squares methods on all F using SHELX-2014 implemented in Olex2. The crystals were mounted on a glass tip using crystallographic oil and placed in a cryostream. Data were collected using φ and ω scans chosen to give a complete asymmetric unit. All non-hydrogen atoms were refined anisotropically. Hydrogen atoms were calculated geometrically riding on their parent atoms. Full crystallographic details can be found in CIF format: see the Cambridge Crystallographic Data Centre database (CCDC 2192851-1547623)

C. CASSCF calculations

For the CASSCF-SO electronic structure calculation of **$Et_4N[^{160}Gd(C_{32}H_{16}N_8)_2]$** *OpenMolcas* was employed[2]. CASSCF-SO calculation was performed only on the Gd^{3+} site employing the crystallographic coordinates obtained from the single crystal X-ray structure with no further optimisation. Basis sets from ANO-RCC library were employed with VTZP quality for the gadolinium ion[3–5], while ANO-DK3 were employed for all remaining atoms[6]. The molecular orbitals (MOs) were optimized in state-averaged CASSCF calculations. For this, the active space was defined by the seven 4f electrons in the seven 4f orbitals of Gd^{3+}. Four calculations were performed independently for each possible spin state, where 1 root was included for S = 7/2, 48 roots were included for S = 5/2, 392 roots were for S = 3/2 and 600 roots for S = 1/2 (RASSCF routine). The wavefunctions obtained from these CASSCF calculations were posteriorly mixed by spin orbit coupling, where the state for S = 7/2 states, 48 of the S = 5/2 states, 129 of the S = 3/2 and 113 of the S = 1/2 states were included (RASSI routine[7]). The resulting spin orbit wavefunctions were decomposed into their CF wavefunctions in the $^8S_{7/2}$ basis, employing the SINGLE_ANISO routine[8,9].

D. Fitting and Simulation of µSQUID-EPR data

The fitting and simulation procedure of the µSQUID-EPR is as follows: the experimental data was sampled and stored as a map: (transition label, magnetic induction) ® resonant frequency.

The spectrum of the spin Hamiltonian (Eq.4) can be obtained by standard diagonalization methods using a "home build" software (EasySpin would be a commercial equivalent). The initial parameters B_q^0 were obtained from Eq. 7, 8, 9, while the non-axial parameters were taken to be of the same order of magnitude as the axial ones. The square deviation between the theoretical prediction of the resonance frequency and experimental values, $\chi^2 = \sum(\nu_{exp} - \nu_{SHP})^2$, is minimized in an iterative process using the Marguardt-Levenberg nonlinear algorithm as implemented in Gnuplot.

Supplementary Table 1. CASSCF calculated ligand field B_q^k parameters for $Et_4N[^{160}Gd(C_{32}H_{16}N_8)_2]$.

q	***k***	**CASSCF (GHz)**
2	0	-0.88287
2	1	-1.79E-06
2	2	2.69E-04
4	0	-2.43E-04
4	1	4.92E-08
4	2	3.17E-08
4	3	-1.50E-08
4	4	-2.32E-09
6	0	-9.31E-09
6	1	-4.97E-11
6	2	-5.97E-12
6	3	4.87E-11
6	4	-1.77E-09
6	5	6.92E-11
6	6	-3.43E-10

Supplementary Table 2. Anisotropic parameters and associated errors.

**Final set of parameters (GHz)**		**Asymptotic Standard Error**	
B_2^0	-6.80×10^{-1}	$\pm2.01\times10^{-3}$	0.2959%
B_2^2	-2.75×10^{-1}	$\pm1.12\times10^{-2}$	4.09%
B_4^0	-1.57×10^{-3}	$\pm3.41\times10^{-5}$	2.175%
B_4^4	3.38×10^{-3}	$\pm4.37\times10^{-5}$	12.92%
B_6^0	1.60×10^{-7}	$\pm5.48\times10^{-8}$	34.22%

Supplementary Table 3. Correlation matrix of the fit parameters.

	B_2^0	B_2^2	B_4^0	B_4^4	B_6^0
B_2^0	1.00				
B_2^2	0.676	1.000			
B_4^0	-0.436	-0.191	1.000		
B_4^4	-0.340	-0.377	0.251	1.000	
B_6^0	0.188	0.113	-0.436	-0.043	1.000

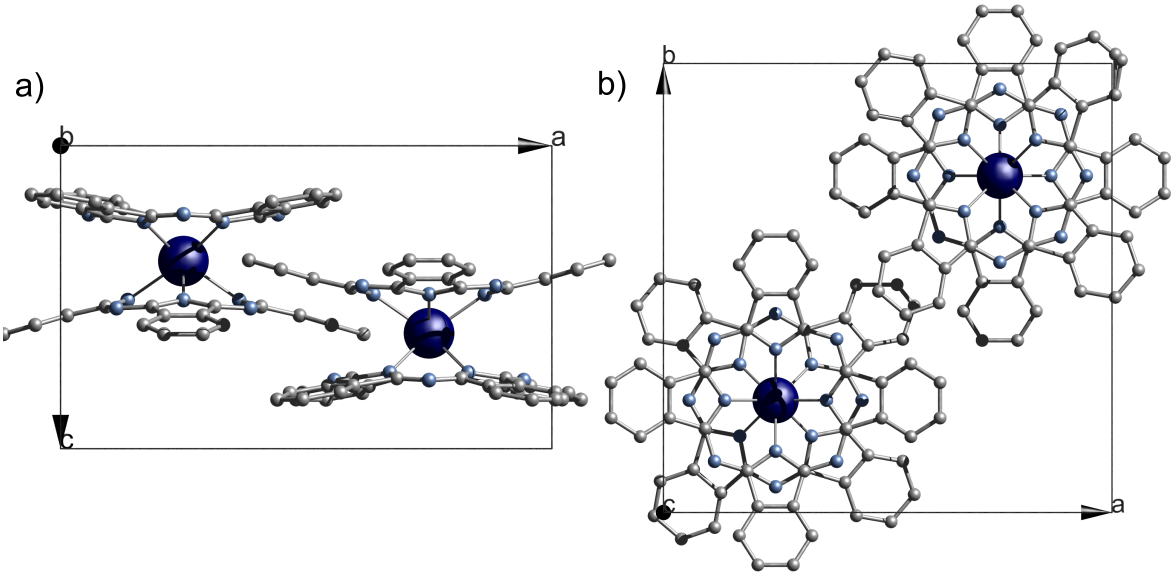

Supplementary Figure 1. a) Side view and b) top view of the unit cell of **$Et_4N[^{160}GdPc_2]$** showing the two molecules residing in the unit cell. Hydrogen atoms have been removed for clarity. (Colour code: Gd, dark blue; O, red; N, cyan; C, grey.

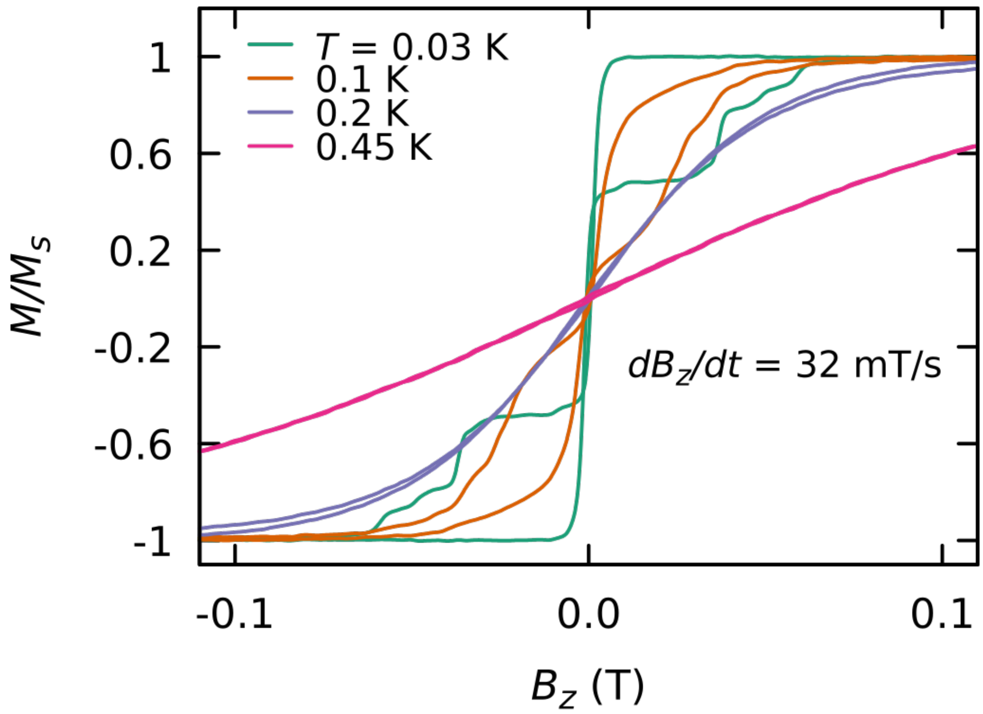

Supplementary Figure 2. Temperature dependence of the magnetisation curves at a fixed sweeping rate of 32 mT/s. Note: The slight opening at high fields observed for the loops at 0.2 K is an artifact of data treatment and not anisotropy.

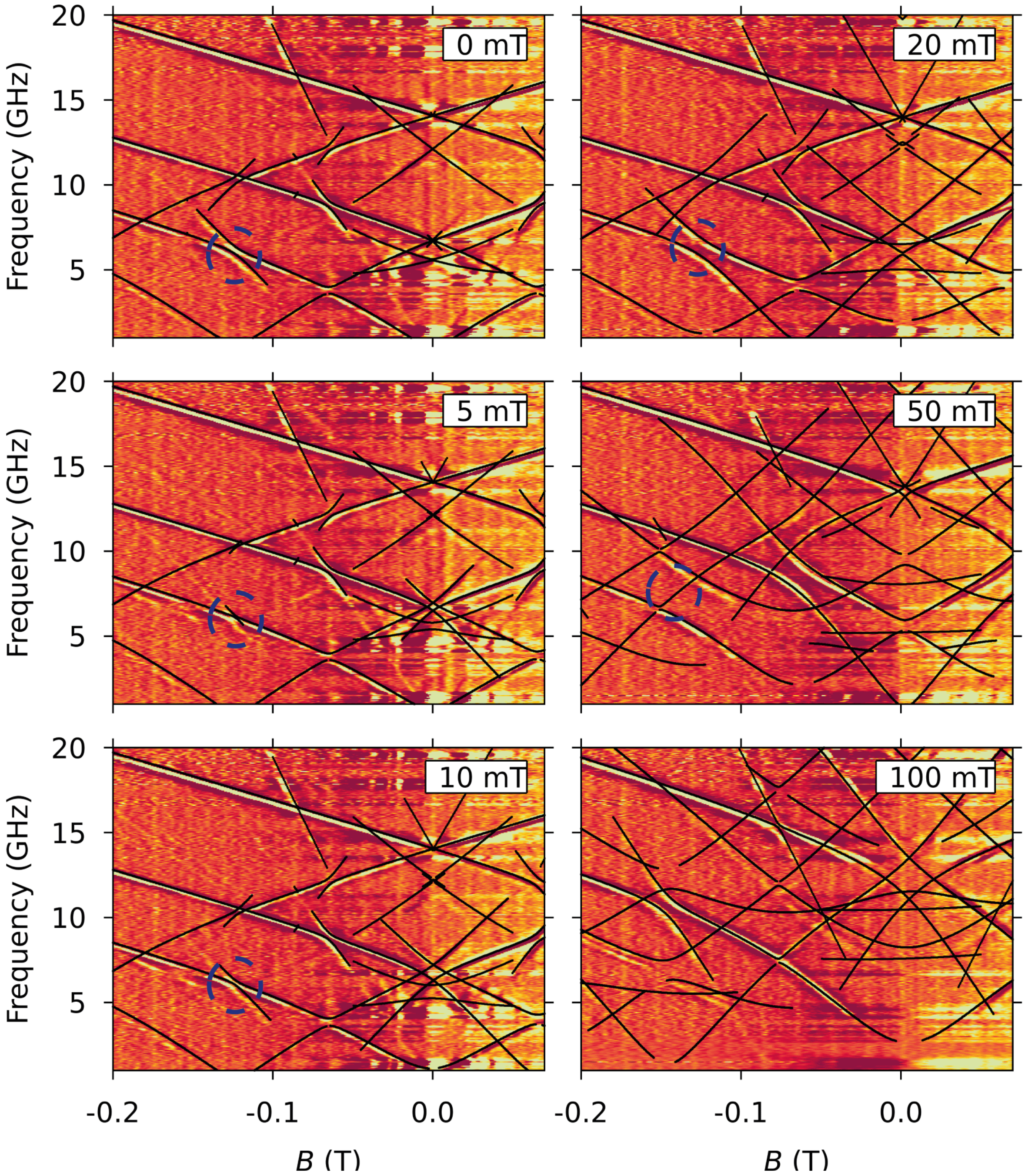

Supplementary Figure 3. Fit of the transverse field maps with g = 1.96, φ = 3◦, B_2^0 = −6.80 × 10^{-1} GHz, B_4^0 = −1.57×10^{-3} GHz, B_6^0 = 1.6×10^{-7} GHz, B_2^2 = −2.75×10^{-1} GHz and B_2^4 = 3.38×10^{-3} GHz. The fit lines were shifted upwards by 20 MHz to better visualize the experimental transitions. Blue circles indicate the oscillations of $\Delta_{-5/2}^{1/2}$ as a function of the applied transverse field bearing evidence to the phase interference effect.

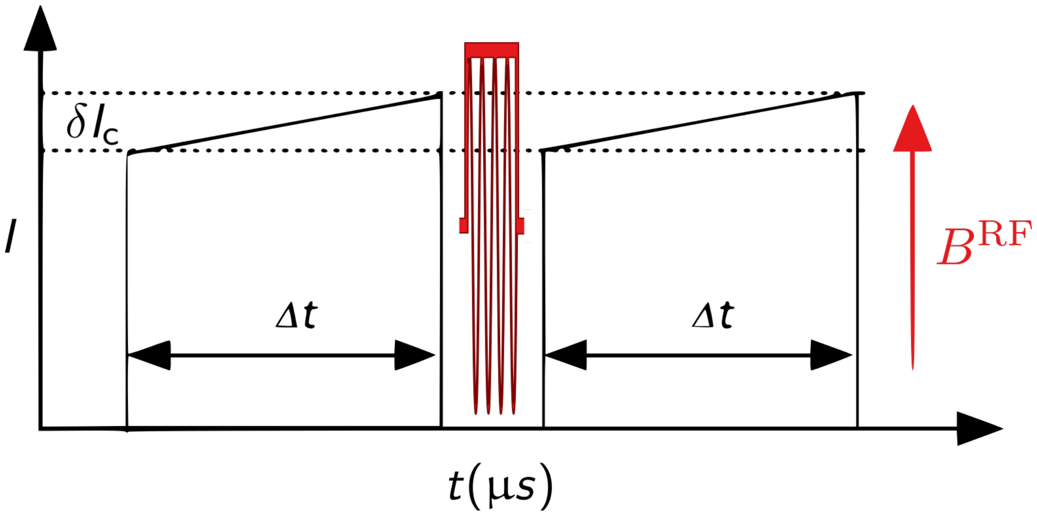

Supplementary Figure 4. Timing of the SQUID measurement and the application of RF pulses to the sample. During the μSQUID-EPR experiments, 40 μs-wide RF pulses (in red) are sent to the sample interleaved with the μSQUID measurement DC current pulses (in black). In order for the μSQUID to perform correctly, the two kinds of pulses should not overlap. The repetition period is 300 μs.

Supplementary References:

1 L. L. Quill, R. F. Fobey and S. Seifter, *Ind. Eng. Chem. Anal. Ed.*, 1937, **9**, 389–392.

2 I. Fdez. Galván, M. Vacher, A. Alavi, C. Angeli, F. Aquilante, J. Autschbach, J. J. Bao, S. I. Bokarev, N. A. Bogdanov, R. K. Carlson, L. F. Chibotaru, J. Creutzberg, N. Dattani, M. G. Delcey, S. S. Dong, A. Dreuw, L. Freitag, L. M. Frutos, L. Gagliardi, F. Gendron, A. Giussani, L. González, G. Grell, M. Guo, C. E. Hoyer, M. Johansson, S. Keller, S. Knecht, G. Kovačević, E. Källman, G. Li Manni, M. Lundberg, Y. Ma, S. Mai, J. P. Malhado, P. Å. Malmqvist, P. Marquetand, S. A. Mewes, J. Norell, M. Olivucci, M. Oppel, Q. M. Phung, K. Pierloot, F. Plasser, M. Reiher, A. M. Sand, I. Schapiro, P. Sharma, C. J. Stein, L. K. Sørensen, D. G. Truhlar, M. Ugandi, L. Ungur, A. Valentini, S. Vancoillie, V. Veryazov, O. Weser, T. A. Wesołowski, P. O. Widmark, S. Wouters, A. Zech, J. P. Zobel and R. Lindh, *J. Chem. Theory Comput.*, 2019, **15**, 5925–5964.

3 B. O. Roos, R. Lindh, P.-Å. Malmqvist, V. Veryazov, P.-O. Widmark and A. C. Borin, *J. Phys. Chem. A*, 2008, **112**, 11431–11435.

4 B. O. Roos, R. Lindh, P. Å. Malmqvist, V. Veryazov and P. O. Widmark, *J. Phys. Chem. A*, 2004, **108**, 2851–2858.

5 B. O. Roos, R. Lindh, P.-Å. Malmqvist, V. Veryazov and P.-O. Widmark, *J. Phys. Chem. A*, 2005, **109**, 6575–6579.

6 T. Tsuchiya, M. Abe, T. Nakajima and K. Hirao, *J. Chem. Phys.*, 2001, **115**, 4463–4472.

7 P. Å. Malmqvist, B. O. Roos and B. Schimmelpfennig, *Chem. Phys. Lett.*, 2002, **357**, 230–240.

8 L. Ungur and L. F. Chibotaru, *Chem. - A Eur. J.*, 2017, **23**, 3708–3718.

9 L. F. Chibotaru and L. Ungur, *J. Chem. Phys.*, 2012, **137**, 064112.
